# Inhibition of Serine Palmitoyl Transferase I Reduces Cardiac Ceramide Levels and Increases Glycolysis Rates following Diet-Induced Insulin Resistance

**DOI:** 10.1371/journal.pone.0037703

**Published:** 2012-05-22

**Authors:** John R. Ussher, Clifford D. L. Folmes, Wendy Keung, Natasha Fillmore, Jagdip S. Jaswal, Virgilio J. Cadete, Donna L. Beker, Victoria H. Lam, Liyan Zhang, Gary D. Lopaschuk

**Affiliations:** Cardiovascular Research Centre, Mazankowski Alberta Heart Institute, University of Alberta, Edmonton, Canada; University of Tor Vergata, Italy

## Abstract

**Objective:**

Diet-induced obesity (DIO) leads to an accumulation of intra-myocardial lipid metabolites implicated in causing cardiac insulin resistance and contractile dysfunction. One such metabolite is ceramide, and our aim was to determine the effects of inhibiting *de novo* ceramide synthesis on cardiac function and insulin stimulated glucose utilization in mice subjected to DIO.

**Materials and Methods:**

C57BL/6 mice were fed a low fat diet or subjected to DIO for 12 weeks, and then treated for 4 weeks with either vehicle control or the serine palmitoyl transferase I (SPT I) inhibitor, myriocin. *In vivo* cardiac function was assessed via ultrasound echocardiography, while glucose metabolism was assessed in isolated working hearts.

**Results:**

DIO was not associated with an accumulation of intra-myocardial ceramide, but rather, an accumulation of intra-myocardial DAG (2.63±0.41 vs. 4.80±0.97 nmol/g dry weight). Nonetheless, treatment of DIO mice with myriocin decreased intra-myocardial ceramide levels (50.3±7.7 vs. 26.9±2.7 nmol/g dry weight) and prevented the DIO-associated increase in intra-myocardial DAG levels. Interestingly, although DIO impaired myocardial glycolysis rates (7789±1267 vs. 2671±326 nmol/min/g dry weight), hearts from myriocin treated DIO mice exhibited an increase in glycolysis rates.

**Conclusions:**

Our data reveal that although intra-myocardial ceramide does not accumulate following DIO, inhibition of *de novo* ceramide synthesis nonetheless reduces intra-myocardial ceramide levels and prevents the accumulation of intra-myocardial DAG. These effects improved the DIO-associated impairment of cardiac glycolysis rates, suggesting that SPT I inhibition increases cardiac glucose utilization.

## Introduction

Obesity, insulin resistance, and diabetes are rapidly increasing in our society [1], [2], [3], [4], and by 2025 it is estimated that more than 300 million individuals between 20 and 79 years of age will suffer from diabetes [5]. Patients who suffer from these conditions often possess a multitude of other pathologies, including hyperlipidemia and hypertension. This set of risk factors is often referred to as the “Metabolic Syndrome” and increases one’s risk of cardiovascular disease, the leading cause of mortality amongst patients in the diabetic population [1]. Thus, therapeutic strategies aimed at alleviating these conditions have the potential to reduce the burden of cardiovascular disease.

As mentioned, obesity, insulin resistance, and diabetes are often accompanied by a hyperlipidemia, which manifests itself in the form of an elevation in plasma free fatty acids (FFAs). While there is no argument that elevated plasma FFAs lead to increased rates of fatty acid uptake in the heart, controversy remains as to how this fatty acid overload contributes to cardiac dysfunction. It has been postulated that an impaired ability of the heart to oxidize this fatty acid surplus leads to an accumulation of intra-myocardial fatty acid metabolites, such as triacylglycerol (TAG), long chain acyl CoA, ceramide, and diacylglyerol (DAG), which contribute to the development of contractile dysfunction, a term coined “cardiac lipotoxicity” [1], [6], [7], [8], [9].

Supporting this proposal, recent findings in the obese Zucker rat demonstrate that cardiac dysfunction is associated with reduced rates of myocardial fatty acid oxidation compared to lean controls during fasting, an effect accompanied by elevated levels of intra-myocardial lipid and an inability to increase the expression of peroxisome proliferator activated receptor α (PPARα) target genes [1]. Conversely, our results show no difference in the rates of myocardial fatty acid oxidation between insulin resistant JCR:LA-cp rats and lean controls during either fasting or ad-libitum conditions [10]. Furthermore, insulin resistant JCR:LA-cp rats showed nearly a doubling in intra-myocardial TAG, suggesting that the accumulation of intra-myocardial fatty acid metabolites is the result of an excessive fatty acid supply, rather than impaired fatty acid oxidation. In addition, we have demonstrated that myocardial fatty acid oxidation rates are increased in transgenic PPARα overexpressing mice, a strain possessing a phenotype resembling that of type 2 diabetes [11]. More recently, we have shown in malonyl CoA decarboxylase deficient mice, a genetic model of reduced fatty acid oxidation rates, a clear disconnect between intra-myocardial TAG levels, insulin sensitivity and cardiac function [12].

Therefore, debate still exists with regards to how the hyperlipidemia observed during obesity, insulin resistance, and diabetes contributes to cardiac lipotoxicity and cardiac dysfunction. As recent work has suggested that an elevation in intra-myocardial ceramide levels may cause lipotoxicity by increasing rates of apoptosis in the heart [13], [14], [15], we investigated this controversy by examining the effect of inhibiting *de novo* ceramide synthesis following diet-induced obesity (DIO) and insulin resistance. This was achieved by feeding mice either a low or high fat diet rich in saturated fat, which plays a major role in *de novo* ceramide synthesis through serine palmitoyl transferase I (SPT I). A pharmacological inhibitor of SPT I was utilized to determine if reducing intra-myocardial ceramide levels could prevent cardiac lipotoxicity and improve myocardial insulin sensitivity. We hypothesized that pharmacological inhibition of *de novo* ceramide synthesis would restore glucose utilization in the insulin-resistant heart and improve cardiac function, implicating ceramide as an important mediator responsible for cardiac lipotoxicity observed in obesity and type 2 diabetes.

## Materials and Methods

### Animal Studies

The University of Alberta adheres to the principles for biomedical research involving animals developed by the Council for International Organizations of Medical Sciences and complies with the Canadian Council on Animal Care guidelines. All animal procedures were approved by the University of Alberta Health Sciences Animal Welfare Committee. 8-week old C57BL/6 wild type mice (Charles River) were placed on a standard chow/low fat diet (4% kcal from lard) or high fat diet (60% kcal from lard, Research Diets; D12492) to produce diet-induced obesity (DIO) over a 12-week period. At the end of week 12, animals were treated every other day with the SPT I inhibitor, myriocin (0.5 mg/kg), via intraperitoneal injection for a 4-week period. At the end of the treatment period, animals were sacrificed (12 mg sodium pentobarbital) in the fed state during the dark cycle, and the hearts were subsequently removed and perfused in the working mode as described below. In another study, 6-week-old *db/db* mice and their heterozygous controls (*db/+*) (Jackson Laboratories) were placed on an identical 4-week treatment regimen.

### Isolated Working Heart Perfusions

Immediately after animal sacrifice, hearts were excised and cannulated via the aorta and left atrium. After equilibration in the Langendorff mode, hearts were switched to the working mode and perfused with modified Krebs-Henseleit buffer (KHB) containing 118.5 mM NaCl, 25 mM NaHCO_3_, 4.7 mM KCl, 1.2 mM MgSO_4_, 1.2 mM KH_2_PO_4_, 2.5 mM CaCl_2_, 5 mM [5-^3^H]/[U-^14^C]glucose, 1.2 mM palmitate pre-bound to 3% fatty acid free bovine serum albumin, and 100 µU/mL insulin. Hearts underwent aerobic perfusion for 40 min and rates of glycolysis and glucose oxidation were measured by quantitative collection of ^3^H_2_O and ^14^CO_2_, respectively, as previously described [16], [17]. At the end of the 40 min aerobic perfusion protocol, hearts were immediately frozen in liquid N_2_ and stored at −80°C until used for biochemical analyses.

### Calculation of Proton (H^+^) Production from Glucose Utilization

H^+^ production attributable to glucose metabolism was calculated from the measured rates of glycolysis and glucose oxidation. If the rate of glycolysis exceeds subsequent glucose oxidation, a net production of two H^+^s per molecule of glucose occurs [18], [19]. Thus, overall rates of H^+^ production derived from glucose utilization were determined by subtracting the rate of glucose oxidation from the rate of glycolysis and multiplying by two.

### *In vivo* Cardiac Function via Echocardiography

*In vivo* cardiac function (ejection fraction, fractional shortening) and M-mode ventricular wall measurements (left ventricular wall thickness and diameters) were assessed in isoflurane anaesthetized mice with a Vevo 770 high-resolution echocardiography imaging system equipped with a 30-MHz transducer (RMV-707B; VisualSonics, Toronto, Canada) as previously described [20].

### Intra-myocardial Fatty Acid Metabolite Determination

Extraction and quantification of long chain acyl CoA and short chain acyl CoA esters via high performance liquid chromatography (HPLC) were performed as previously described [10], [21]. TAGs were extracted with a 2∶1 chloroform-methanol solution and quantified with a commercially available enzymatic assay kit (Wako Pure Chemical Industries) as previously described [10]. Ceramides were extracted via HPLC, and DAGs were extracted via thin layer chromatography (TLC) as previously described [22]. In brief, myocardial tissue was extracted for ceramide with 1 mL of a 1∶1:1 chloroform-methanol-1 *N* HCl in the presence of 0.3 mL saline solution. The resulting organic phase was separated and dried under N_2_. 0.5 mL of 1 M KOH in 90% (v/v) methanol was added and samples were heated at 90°C for 1 hr to deacylate ceramide into sphingosine. Samples are then extracted with 1 M HCl in methanol, chloroform, and 1 M aqueous NaCl. The resulting organic phase was dried under N_2_, re-dissolved in methanol, and derivatized to *o*-Phthalaldehyde to generate a fluorescent compound that was separated by HPLC and quantified by fluorescence spectrometry. With regards to DAG, ∼5 mg of myocardial tissue was homogenized in 0.8 ml of 1 mM NaCl. The homogenate was then transferred over to a 13 x 100 mm test tube and extracted with 3.0 ml of 1∶2 chloroform-methanol and mixed. 1 ml of 1 mM NaCl and chloroform was then added to break phases and the resulting sample was centrifuged briefly at 5,000 x*g* to separate the organic and aqueous phases. The chloroform phase was then collected and evaporated under N_2_ gas. The lipids were then solubilized with a 7.5% octyl-β-D-glucoside/5mM cardiolipin/1mM DETAPAC solution and incubated at room temperature for 5 to 15 min. The sample was then brought to 100 µl volume with 50 µl of 2x reaction buffer, 20 µl 10 mM DTT and 10 µl DAG kinase (0.5 mg/ml). The reaction was initiated by the addition of 10 µl of 10 mM ATP (mixed with [γ-^32^P]ATP) and incubated for 30 min at 25°C. 3 mL chloroform/methanol (1∶2) and 0.7 mL 1% HClO_4_ was then added to each tube and mixed before the addition of 1 mL chloroform and 1 mL 1% HClO_4_. The phases were then separated via centrifugation at 2,000 x*g* at 4°C for 5 min. The aqueous phase was discarded and the chloroform phase washed twice with 2 ml of 1% HClO_4_. The sample was then evaporated under N_2_ gas and dissolved in 100 µl of 5% methanol in chloroform. 20 µl of each sample was spotted onto a TLC plate followed by activation with acetone (develop with choloroform/methanol/acetic acid (65:15:5)). After TLC plate has ran, the plate was exposed to X-ray film and developed for ∼48 hrs. Finally, the silicon was scraped off the plate into 5 ml scintillation vials with 4 ml of scintillation solution (Ecolite, ICN) and ^32^P counted in a liquid scintillation counter.

### Pyruvate Dehydrogenase (PDH) Activity

PDH activities were measured using a revised protocol based on the radiometric assay described by Constantin-Teodosiu *et al.* [23], and as previously described [12]. Briefly, for measurement of active PDH, frozen myocardial tissue (∼20 mg) was homogenized in buffer containing 200 mM sucrose, 50 mM KCl, 5 mM EGTA, 50 mM Tris HCl, 50 mM NaF, 50 mM sodium pyrophosphate (NaPPi), 5 mM dicholoroacetate, and 0.1% Triton X-100 (pH 7.8). For assay of “total” PDH activity (dephosphorylated), frozen tissue was homogenized in buffer containing 1 mM CaCl_2_ but in the absence of NaF, NaPPi, and EGTA. Samples were neutralized and centrifuged, and the resulting supernatant was used for determination of acetyl-CoA content. Acetyl CoA was converted to [^14^C]citrate and separated from unreacted radioactivity using Dowex resin (50WX8, 100–200 mesh). The amount of acetyl CoA was determined by comparison of acetyl CoA standard curves ran in parallel during the experiment.

### Hydroxyacyl CoA Dehydrogenase Activity

Frozen ventricular tissue (5–10 mg) was homogenized in buffer containing 50 mM Tris HCl (pH 8 at 4°C), 1 mM EDTA, 10% glycerol (w/v), 0.02% Brij-35 (w/v), 1 mM DTT, protease and phosphatase inhibitors (Sigma). After homogenization for 30 sec, the homogenate was left on ice for 10 min before centrifugation at 10,000 x *g* for 20 min. The resulting supernatant was brought to a final dilution of 1/20, and 10 µL of each sample was pipetted into a 96 well plate. Each well was brought to a final volume of 190 µL with 160 µL of 50 mM imidazole (pH 7.4) and 20 µL of 1.5 mM NADH. The reaction was then initiated by the addition of 10 µL of 2 mM acetoacetyl CoA and the reaction mixture has its absorbance followed at a 340 nM wavelength for a 5 min duration (readings taken in 30 sec intervals) with a spectrophotometer kinetic plate reader. The 96 well plate was read at a 340 nM wavelength for a 5 min duration as well before the addition of acetoacetyl CoA in order to obtain a baseline reading. The final reading was multiplied by 60 to obtain a rate per min, and hydroxyacyl CoA Dehydrogenase activity in “µmol/min/g wet weight” was calculated with the following equation:
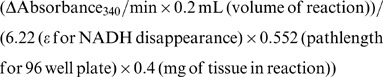



### Immunoblot Analysis

Immunoblots were carried out to determine protein expression and phosphorylation in hearts as previously described [24]. In brief, frozen ventricular tissue (25–30 mg) was homogenized in buffer containing 50 mM Tris HCl (pH 8 at 4°C), 1 mM EDTA, 10% glycerol (wt/vol), 0.02% Brij-35 (wt/vol), 1 mM dithiothreitol, protease and phosphatase inhibitors (Sigma). After homogenization for 30 s, the homogenate was left on ice for 10 min before centrifugation at 10 000 x *g* for 20 min. The resulting supernatant was processed for immunoblotting. Protein concentration of homogenates was determined via Bradford protein assay kit (Bio-Rad). Samples were resolved via 8% sodium didecyl sulfate polyacrylamide gel electrophoresis (SDS-PAGE) and transferred onto a 0.45 µm nitrocellulose membrane. Membranes were blocked with 10% fat free milk for 2 hours and probed with either anti-AMPK (Cell Signaling Technologies, 1/1000 dilution), anti-phosphoThreonine-172 AMPK (Cell Signaling Technologies, 1/500 dilution), anti-Akt (Cell Signaling Technologies, 1/1000 dilution), anti-phosphoSerine-473 Akt (Cell Signaling Technologies, 1/500 dilution), anti-GSK3β (Cell Signaling Technologies, 1/1000 dilution), anti-phosphoSerine-9 GSK3β (Cell Signaling Technologies, 1/500 dilution), anti-acetyl CoA carboxylase (ACC, Jackson ImmunoResearch Laboratories, 1/1000 dilution), anti-peroxisome proliferator activated receptor alpha (PPARα, Abcam, 1/1000 dilution), anti-CD36 (Santa Cruz Biotechnology, 1/1500 dilution), anti-uncoupling protein 3 (UCP3, Alpha Diagnostic, 1/2000 dilution), and anti-pyruvate dehydrogenase kinase 4 (PDK4, Abgent, 1/200 dilution) antibodies in 5% fatty acid free bovine serum albumin. Immunoblots were visualized with the enhanced chemiluminescence Western blot detection kit (Perkin Elmer) and quantified with Quantity One (4.4.0) Software (Biorad Laboratories).

### Statistical Analyses

All values are presented as mean ± SE (*n* observations). The significance of differences was determined by the use of an unpaired, two-tailed Student’s *t*-test or one-way analysis of variance (ANOVA), followed by Bonferroni’s post-hoc analysis where appropriate. Differences were considered significant when *P*<0.05.

## Results

### Diet-induced Obesity and Subsequent Insulin Resistance do not Cause Cardiac Dysfunction

**Figure 1:**
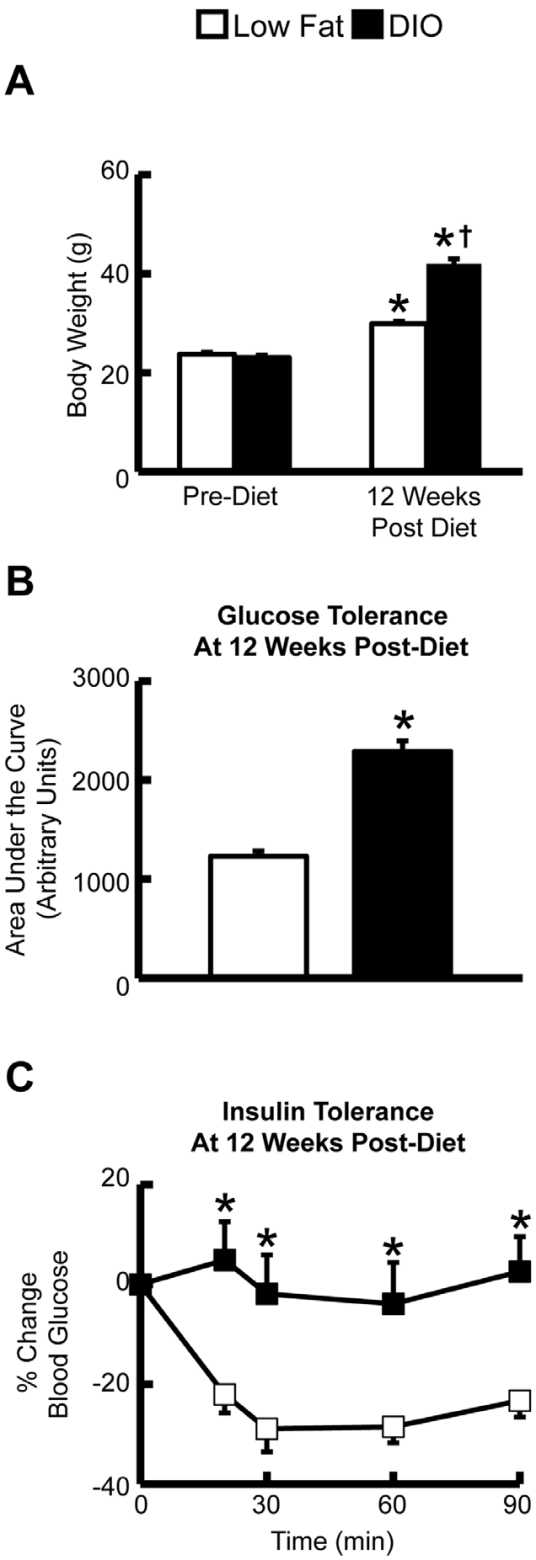
Insulin resistance in mice following DIO. *A:* 12 weeks of DIO results in significant increases in body weight in mice. *B:* This increase in body weight resulted in whole body glucose intolerance, and *C:* insulin resistance. Values represent mean ± SE (n = 10–11). Glucose and insulin were administered via intraperitoneal injection. Differences were determined using an unpaired Student’s two-tailed t-test or two-way ANOVA followed by a Bonferroni’s post-hoc analysis. *P<0.05, significantly different from low fat fed counterpart. ^†^ *P*<0.05, significantly different from low fat fed counterpart at 12 weeks post-diet.

Following 12 weeks of high fat feeding, animals became severely obese and insulin resistant (Figure 1). Interestingly, this was not accompanied by any cardiac dysfunction or hypertrophy as determined via ultrasound echocardiography (Table 1). Similar results were obtained when hearts were aerobically perfused in the working mode for a 40 min period after 16 weeks of high fat feeding (Table 2).

**Table 1:** *In vivo* cardiac function and left ventricular wall measurements in lean and DIO mice treated with myriocin.

StudyGroup	LeanControl(n = 4)	LeanMyriocin(n = 5)	DIOControl(n = 5)	DIOMyriocin(n = 6)
EF (%)	62.9±4.1	64.2±1.5	59.2±2.5	58.7±2.3
FS (%)	33.8±2.8	34.5±1.1	31.3±1.7	30.8±1.5
IVSd (mm)	0.83±0.02	0.78±0.01	0.86±0.03	0.84±0.02
LVIDd (mm)	4.16±0.14	3.97±0.05	4.30±0.09	4.24±0.15
LVPWd (mm)	0.78±0.02	0.77±0.03	0.83±0.03	0.83±0.02
IVSs (mm)	1.20±0.04	1.14±0.04	1.28±0.03	1.22±0.04
LVIDs (mm)	2.76±0.20	2.60±0.07	2.96±0.11	2.94±0.15
LVPWs (mm)	1.21±0.03	1.18±0.02	1.24±0.06	1.22±0.05

*In vivo* cardiac function and ventricular wall measurements were assessed via echocardiography in isoflurane anesthetized lean or DIO mice treated with vehicle control or myriocin. Values represent means ± SE. *P<0.05, indicates a significant difference from control treated mice. LVPW = left ventricular posterior wall, LVID = left ventricular internal diameter, IVS = intraventricular septum, d = diastole, s = systole.

**Table 2:** *Ex vivo* cardiac function in lean and obese mice treated with myriocin.

StudyGroup	LeanControl(n = 7)	LeanMyriocin(n = 7)	DIOControl(n = 6)	DIOMyriocin(n = 7)
Heart Rate(beats per minute)	269.9±16.4	284.4±19.3	262.1±10.2	246.4±11.2
Cardiac Output(mL/min)	12.5±0.4	11.0±1.0	10.9±0.4	9.2±0.6
Cardiac Work(mL*mmHg/min)	8.2±0.4	7.2±0.8	7.6±0.3	7.0±0.5

Parameters of cardiac function were assessed in isolated working hearts obtained from mice subjected to either a low fat or high fat diet and treated with vehicle control or myriocin (n = 6–7). Values represent means ± SE.

### Diet-induced Obesity and Insulin Resistance Reduce Myocardial Glycolysis Rates, Which are Restored by Myriocin Treatment

**Figure 2:**
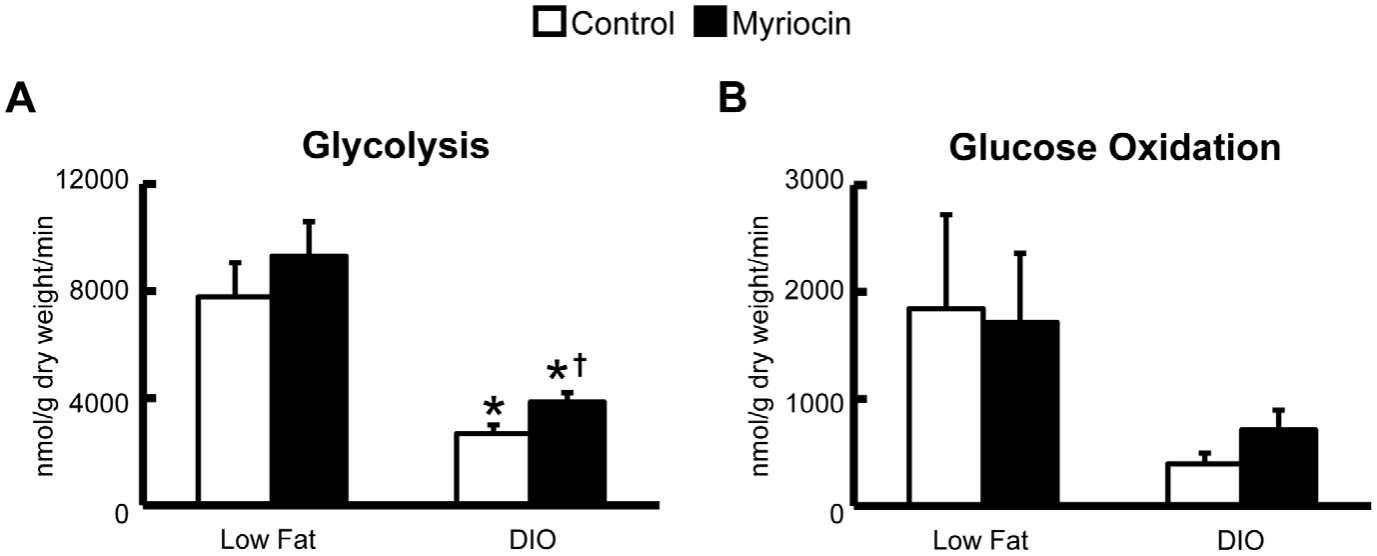
SPT I inhibition improves myocardial glycolysis rates in DIO mice. *A:* DIO impairs insulin-stimulated glycolysis rates in hearts from control treated mice, which was improved in hearts from myriocin treated DIO mice. *B:* Rates of glucose oxidation in hearts from control and myriocin treated low fat fed and DIO mice. Values represent mean ± SE (n = 6–7). Differences were determined using a one-way ANOVA followed by a Bonferroni’s post-hoc analysis. *P<0.05, significantly different from low fat fed counterpart. ^†^ *P*<0.05, significantly different from DIO control.

**Figure 3:**
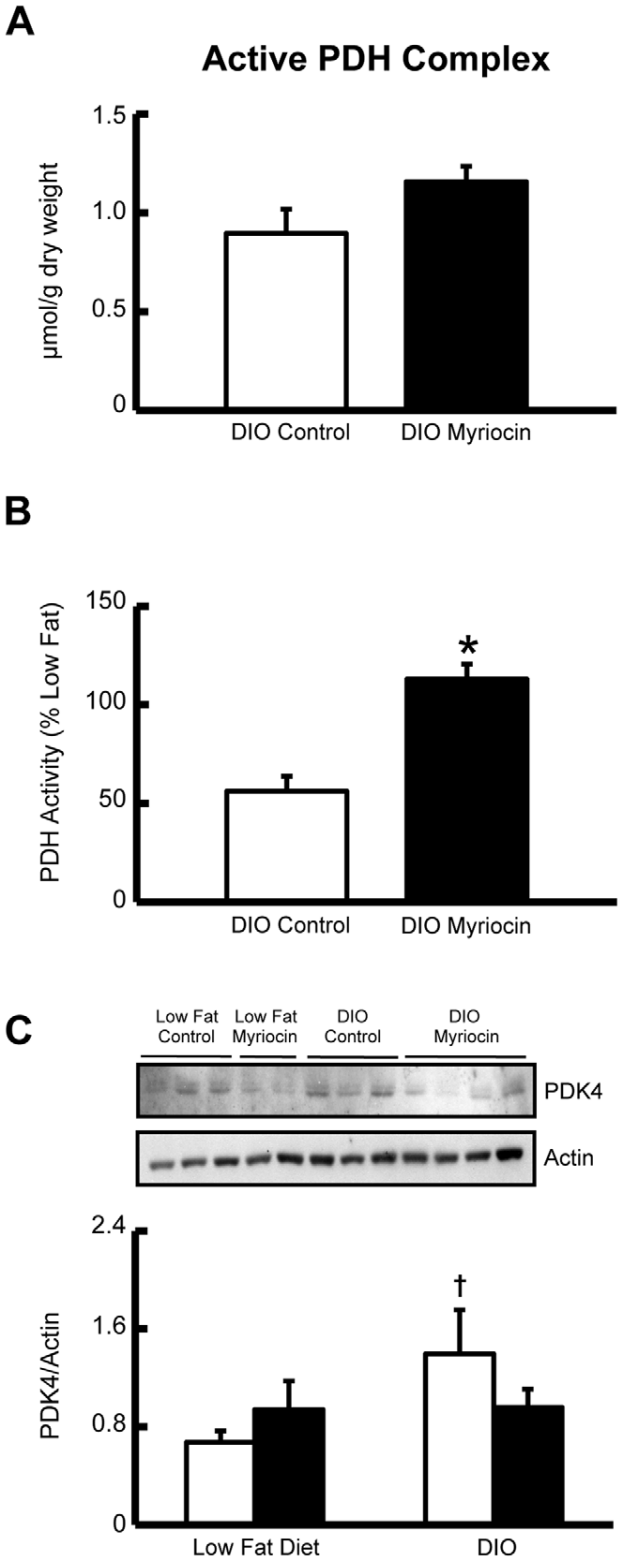
Cardiac PDH activity is not impaired in DIO mice treated with myriocin. *A:* Cardiac PDH activity in control and myriocin treated DIO Mice. *B:* % decrease in cardiac PDH activity relative to low fat fed counterparts. *C*: Cardiac PDK4 protein expression is increased in control treated DIO mice, but not myriocin treated DIO mice. Values represent mean ± SE (n = 6). Differences were determined using an unpaired Student’s two-tailed t-test or a one-way ANOVA followed by a Bonferroni’s post-hoc analysis. *P<0.05, significantly different from DIO control mice. ^†^ *P*<0.05, significantly different from low fat fed counterpart.

**Figure 4:**
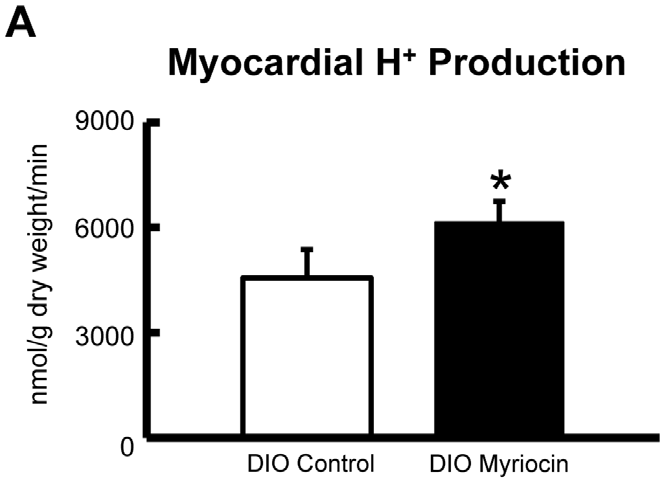
H^+^ production in hearts from DIO mice. H^+^ production was calculated in control and myriocin treated DIO mice by subtracting the rate of glucose oxidation from the rate of glycolysis and multiplying by two. Values represent mean ± SE (n = 6–7). Differences were determined using an unpaired Student’s two-tailed t-test. *P = 0.13.

Aerobic perfusion of hearts following DIO showed a marked reduction in myocardial glycolysis rates, and a strong trend towards a reduction in glucose oxidation rates (Figure 2). However treatment with the SPT I inhibitor, myriocin (0.5 mg/kg), every other day for 4 weeks increased myocardial glycolysis rates in DIO mice compared to control treated DIO mice (Figure 2A). In addition, a trend to increased myocardial glucose oxidation rates was observed in DIO myriocin treated mice (Figure 2B). Further support for this was evident by the fact that myocardial PDH activity was reduced by DIO in the control treated mice, but not in the myriocin treated mice (Figure 3A/B). Moreover, protein expression of PDK4, the kinase responsible for phosphorylating and subsequently inhibiting PDH, was increased in DIO control treated mice versus their lean counterparts, but not in the DIO myriocin treated mice versus their lean counterparts (Figure 3C). Interestingly, we observed a small trend to an increased H^+^ production from uncoupled glucose metabolism in hearts from DIO mice treated with myriocin (Figure 4).

### Myriocin Treatment does not Affect Malonyl CoA Content or Fatty Acid Oxidation Protein Expression Following Diet-induced Obesity and Insulin Resistance

**Figure 5:**
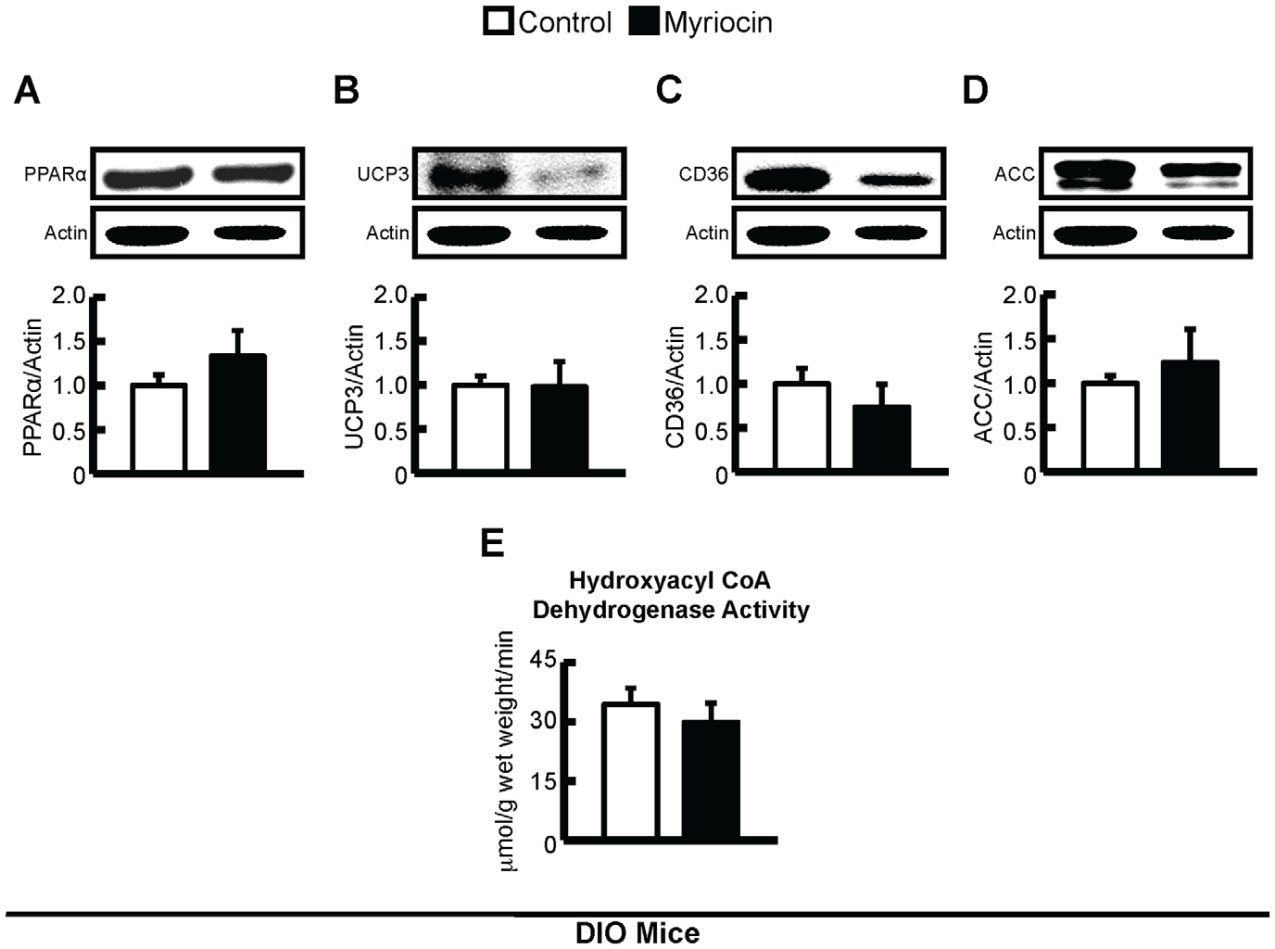
Expression of enzymes involved in the regulation of fatty acid oxidation and hydroxyacyl CoA dehydrogenase activity in DIO mice. Protein expression of *A:* PPARα, *B:* UCP3, *C:* CD36, and *D:* ACC in hearts from control and myriocin treated DIO mice. *E:* Hydroxyacyl CoA dehydrogenase activity was similar in ventricular homogenates from control and myriocin treated DIO mice. Values represent mean ± SE (n = 5–7).

Although rates of myocardial fatty acid oxidation were not measured in this study, levels of malonyl CoA, a major determinant of fatty acid oxidation rates [25], [26], were not altered by either high fat feeding or myriocin treatment, suggesting that fatty acid oxidation rates were not different between groups (Table 3). Furthermore, protein expression of PPARα or its downstream targets, UCP3 and CD36, were similar in hearts from control and myriocin treated DIO mice, as was the expression of ACC (Figure 5A–D), the key enzyme involved in the generation of malonyl CoA. No change in the activity of the mitochondrial fatty acid oxidation enzyme, hydroxyacyl CoA dehydrogenase, was observed in hearts from control and myriocin treated DIO mice (Figure 5E). Plasma fatty acid delivery to the heart can also regulate myocardial fatty acid oxidation rates, and measurement of plasma lipids demonstrated no difference in circulating fatty acids between DIO control and myriocin treated mice, though a trend to reduced circulating TAGs was observed in myriocin treated mice (Table 4).

**Table 3:** Cardiac malonyl CoA levels (nmol/g dry weight).

Study Group	Lean Control	Lean Myriocin	DIO Control	DIO Myriocin
Malonyl CoA	4.46±0.72	5.77±1.17	6.96±1.02	9.49±2.31

Cardiac malonyl CoA content was measured in WT mice fed a low or high fat diet and treated with vehicle control or myriocin (n = 5–6). Values represent means ± SE.

**Table 4:** Plasma Lipids in DIO and *db/db* mice.

StudyGroup	DIOControl	DIOMyriocin	*db/db*Control	*db/db*Myriocin
TAG (mg/dL)	53±11	34±6	244±42	165±26
FFA (mM)	0.44±0.05	0.48±0.06	0.71±0.09	0.70±0.06

Plasma TAG and FFA levels ad libitum were measured in DIO and *db/db* mice treated with vehicle control or myriocin (n = 4–7). Values represent means ± SE.

### Myriocin Treatment Improves Myocardial Akt Signaling Following Diet-induced Obesity and Insulin Resistance

**Figure 6:**
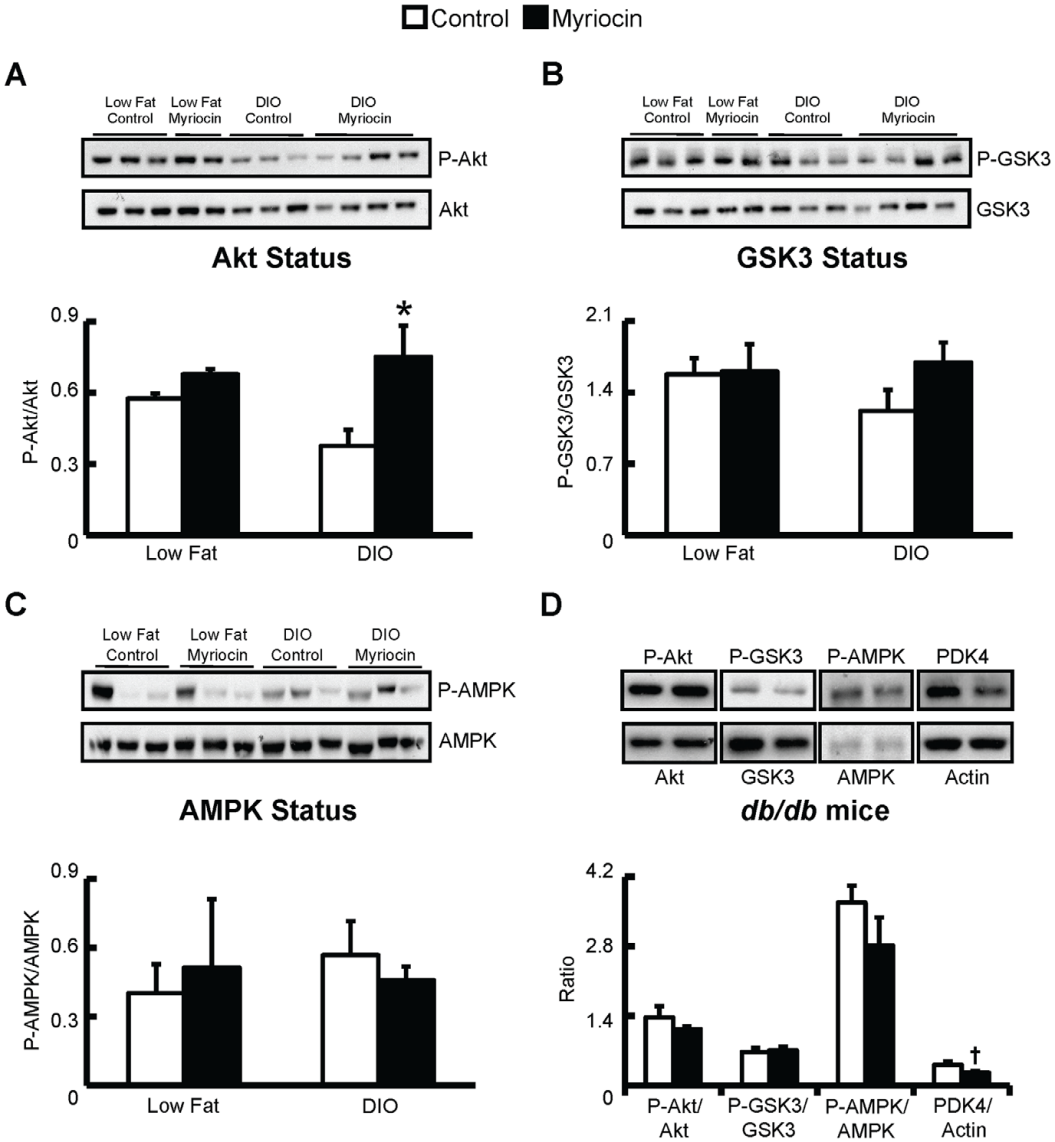
Cardiac Akt phosphorylation in increased in myriocin treated DIO mice. Isolated working mouse hearts were perfused aerobically in the presence of insulin (100 µU/mL ) for determination of, *A:* Cardiac Akt serine 473 phosphorylation, *B:* GSK3β serine 9 phosphorylation, and *C:* AMPK threonine 172 phosphorylation in low fat fed and DIO mice treated with control or myriocin *D:* Cardiac Akt serine 473 phosphorylation, GSK3β serine 9 phosphorylation, AMPK threonine 172 phosphorylation, and PDK4 expression in control and myriocin treated *db/db* mouse hearts. Values represent mean ± SE (n = 4–7). Differences were determined using an unpaired Student’s two-tailed t-test or a one-way ANOVA followed by Bonferroni post-hoc analysis. *P<0.05, significantly different from DIO control treated mice. ^†^ *P* = 0.06.

In parallel to the restoration of myocardial glycolysis rates, we observed increased phorphorylation of Akt at serine 473, which is indicative of Akt activity, in hearts from DIO mice treated with myriocin compared to vehicle control (Figure 6A). However, phosphorylation of downstream targets of Akt, such as glycogen synthase kinase 3 (GSK3) only demonstrated a trend to an increase in DIO mice treated with myriocin compared to vehicle control (Figure 6B).

### Myriocin Treatment Leads to a Marked Reduction in Intra-myocardial Ceramide Levels and Prevents the Diet-induced Obesity Associated Increase in DAG Levels

**Figure 7:**
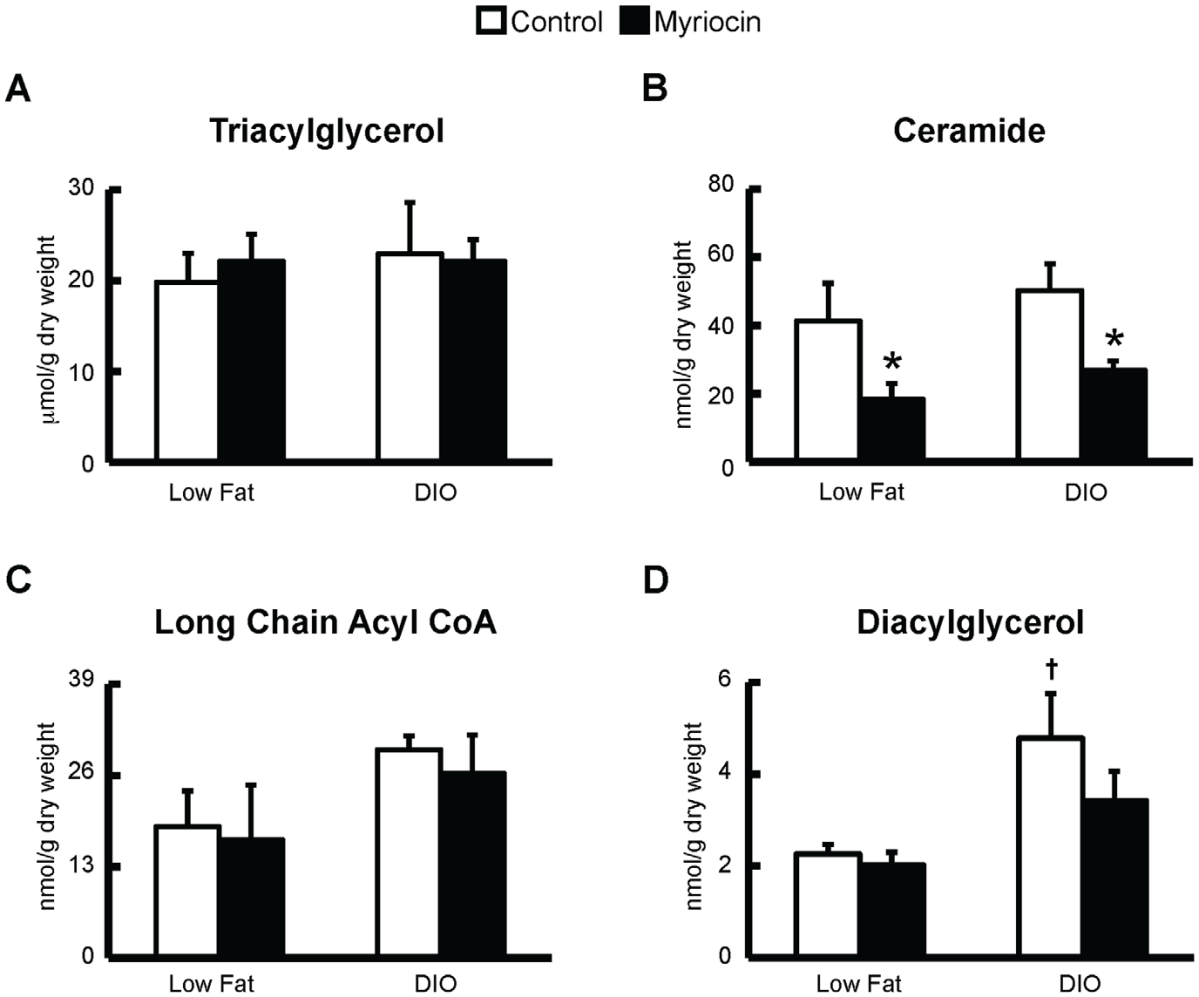
Myriocin treatment of DIO mice decreases intra-myocardial ceramide levels and prevents the accumulation of intra-myocardial DAG. *A:* Intra-myocardial TAG, *B:* ceramide, *C:* long chain acyl CoA, and *D:* DAG levels in low fat fed and DIO mice treated with vehicle control or myriocin. Values represent mean ± SE (n = 4–8). Differences were determined using a one-way ANOVA followed by Bonferroni post-hoc analysis. *P<0.05, significantly different from vehicle control counterpart. ^†^ *P*<0.05, significantly different from low fat fed counterpart.

Similar to what we have reported in previous studies [12], DIO does not increase myocardial TAG levels (Figure 7A). Nonetheless, as expected, inhibition of *de novo* ceramide synthesis at the level of SPT I via myriocin treatment markedly reduced intra-myocardial ceramide levels (Figure 7B). Surprisingly, DIO per se did not actually increase intra-myocardial ceramide levels (Figure 7B), mimicking our previous findings [12], [27]. In contrast to our previous studies [12], [27], we did not observe an increase in intra-myocardial long chain acyl CoAs following DIO (Figure 7C). However, this likely arises from the high palmitate concentration used in the perfusate (1.2 mM), which would arbitrarily increase the long chain acyl CoA levels in perfused hearts from low fat fed animals. Interestingly, DIO increased DAG levels in hearts from control treated mice, and although there was no difference in DAG levels in hearts from myriocin treated DIO mice, DIO did not increase intra-myocardial DAG levels in myriocin treated mice versus their low fat fed counterparts (Figure 7D).

### Myriocin Treatment has no Effect on Cardiac Function or Insulin Signaling in Obese Leptin Receptor Deficient *db/db* Mice

**Figure 8:**
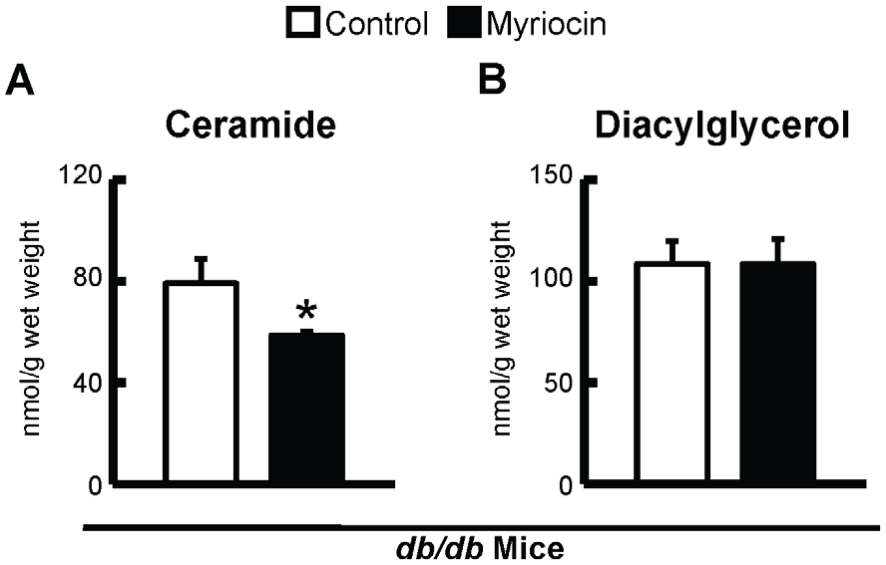
Expression of enzymes involved in the regulation of fatty acid oxidation and hydroxyacyl CoA dehydrogenase activity in *db/db* mice. Protein expression of *A:* PPARα, *B:* UCP3, *C:* CD36, and *D:* ACC in hearts from control and myriocin treated *db/db* mice. *E:* Hydroxyacyl CoA dehydrogenase activity was similar in ventricular homogenates from control and myriocin treated *db/db* mice. Values represent mean ± SE (n = 5–6).

**Figure 9:**
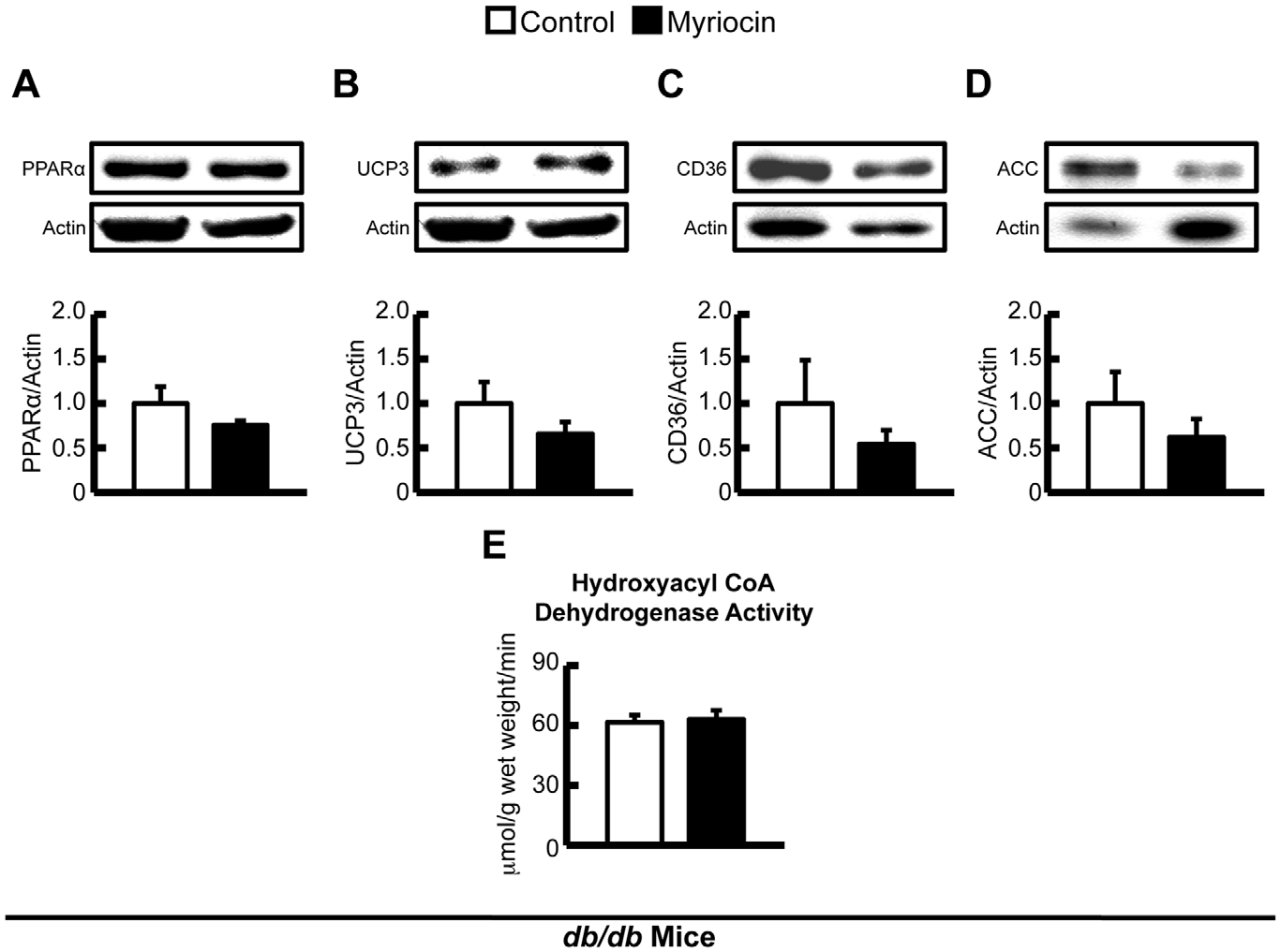
Myriocin treatment of *db/db* mice decreases intra-myocardial ceramide levels but has no effect on intra-myocardial DAG content. *A:* Intra-myocardial ceramide, and *B:* DAG levels in *db/db* mice treated with vehicle control or myriocin. Values represent mean ± SE (n = 4–6). Differences were determined using an unpaired Student’s two-tailed t-test. *P<0.05, significantly different from vehicle control counterpart.

In another set of experiments, obese and insulin resistant *db/db* mice, and their heterozygous controls (*db/+*), were placed on a myriocin treatment regimen identical to the DIO mice. Treatment of *db/db* mice with myriocin decreased intra-myocardial ceramide content, but had no effect on DAG content (Figure 8). Although myriocin treatment prevented the development of insulin resistance and improved skeletal muscle insulin signaling in *db/db* mice cardiac insulin signaling itself was not impaired in *db/db* versus *db/+* mice (data not shown). As such, myriocin treatment had no effect on insulin stimulated myocardial Akt, GSK3, or AMPK phosphorylation in *db/db* mice (Figure 6D), but interestingly, did show a strong trend to a reduction in myocardial PDK4 expression (*P* = 0.06, Figure 4D). As PDK4 negatively regulates PDH activity, and *db/db* mice have reduced myocardial glucose oxidation rates [28], [29], this suggests the possibility that despite myriocin treatment having no effect on insulin signaling in *db/db* mice, it may improve myocardial glucose utilization at the level of oxidation, similar to what is observed in myriocin treated DIO mice. PDK4 expression/activity is increased in response to PPARα and/or enhanced fatty acid oxidation [11]. Measurement of PPARα and its downstream target genes, UCP3 and CD36, revealed a general trend to reduced PPARα signaling in *db/db* myriocin treated mice (Figure 9A–C), while a trend to reduced circulating TAGs was also observed in *db/db* myriocin treated mice (Table 4). This suggests that reduced plasma lipid delivery and subsequent PPARα activity is responsible for the decline in PDK4 expression. Despite these potential metabolic changes, there were no differences in hydroxyacyl CoA dehydrogenase activity between hearts from control and myriocin treated *db/db* mice (Figure 9E), while cardiac function was not impaired in *db/db* mice, and myriocin treatment did not alter function or left ventricular wall measurements (Table 5).

**Table 5:** Cardiac function and left ventricular wall measurements in *db/db* mice treated with myriocin.

Study Group	*db/db* control	*db/db* myriocin
EF (%)	68.1±3.1	63.8±2.5
FS (%)	37.5±2.5	34.2±1.8
LV Mass (mg)	62.0±3.1	68.1±2.4
IVSd (mm)	0.63±0.01	0.68±0.02
LVIDd (mm)	3.75±0.09	3.83±0.11
LVPWd (mm)	0.64±0.02	0.65±0.02
IVSs (mm)	1.05±0.03	1.06±0.03
LVIDs (mm)	2.35±0.12	2.52±0.11
LVPWs (mm)	1.01±0.03	1.00±0.04

*In vivo* cardiac function and ventricular wall measurements were assessed via echocardiography in isoflurane anesthetized *db/db* mice treated with vehicle control or myriocin (n = 5). Values represent means ± SE. LVPW = left ventricular posterior wall, LVID = left ventricular internal diameter, IVS = intraventricular septum, d = diastole, s = systole.

## Discussion

We report in this study that while DIO itself *per se* does not elevate intra-myocardial ceramide levels, inhibition of SPT I to prevent *de novo* synthesis of ceramide results in a marked reduction in overall intra-myocardial ceramide levels, and improves the DIO-associated reduction in myocardial glycolysis rates. Such findings yield provocative insights into the role of obesity on cardiac lipotoxicity and function. It has been proposed that during states of obesity, the accumulation of lipid metabolites in the heart such as TAG, long chain acyl CoA, DAG, and ceramide results in contractile dysfunction and contributes to the development of cardiomyopathy [14], [30]. Ceramide, in particular, is believed to play a major role in the development of cardiac lipotoxicity, as its accumulation has been shown in a number of *in vitro* and *in vivo* models to cause apoptosis of cardiac myocytes, which itself would contribute to the contractile dysfunction associated with cardiac lipotoxicity [13], [14], [15], [31], [32].

In contrast, our findings suggest that DIO does not result in ceramide accumulation in the heart, suggesting that perhaps the ceramide-induced apoptosis, which contributes to cardiac dysfunction, is the result of extreme situations induced by transgene overexpression [13], [14]. Nonetheless, the ceramide/sphingolipid pool is under dynamic regulation, being influenced by both its rates of synthesis and degradation [15]. It is possible that enzymes involved in ceramide degradation were also upregulated by DIO, thereby masking any increase in its synthesis. Studies in humans support this possibility, as the mRNA expression of ceramide metabolizing/degrading enzymes such as ceramidase and sphingosine kinase is increased in right atrial appendages of obese humans with and without a history of type 2 diabetes, explaining why intra-myocardial ceramide content was similar to lean humans despite an elevation in SPT I mRNA expression [33]. Furthermore, our measurement of overall intra-myocardial ceramide content does not separate ceramide originating from *de* *novo* synthesis via SPT I versus that originating from phospholipid hydrolysis.

Our findings differ slightly from a recent study by Park TS *et al.*, who demonstrated that cardiac-specific overexpression of glycosylphosphatidylinositol anchored human lipoprotein lipase in mice is associated with cardiac dysfunction and elevated levels of intra-myocardial ceramide [14]. However, treatment of these animals with myriocin prevented intra-myocardial ceramide accumulation, restored glucose oxidation, and restored cardiac function. Although we also observed improved glucose metabolism in our DIO mice treated with myriocin, we did not observe any form of cardiac dysfunction. It may be possible that our high fat diet model did not result in cardiac dysfunction because intra-myocardial ceramides did not accumulate. A recent study in middle-aged mice (40–44 weeks) has shown that ceramides do accumulate in the heart following a 12-week high fat feeding regimen [34]. However, systolic cardiac function was not altered, suggesting that an elevation of ceramide content in the heart may not negatively impact cardiac function. Nonetheless, future studies should be aimed at investigating the time points required for DIO in mice to induce cardiac dysfunction, and whether manipulation of SPT 1 and ceramide content in the heart can improve this deficit.

In our model, DIO did not result in a rise in intra-myocardial TAG content. While such findings may seem unexpected, we have previously shown that in C57BL/6 mice, intra-myocardial TAG content initially increases following 3 weeks of DIO, but reverts to normal levels seen in lean mice at 10 weeks of DIO, due to a decrease in DGAT activity, contributing to the DIO-associated elevation in DAG content [27]. This is consistent with the observations of Somoza *et al.*, as they demonstrated an increase in intra-myocardial TAG content at 4 weeks of DIO in C57BL/6J mice, followed by a reversion to levels seen in lean mice after 8 weeks of DIO [35]. Contrary to our previous observations, DIO also did not increase intra-myocardial long chain acyl CoA levels [12], [27]. It is important to note that this observation is likely due to the high palmitate concentration used in the perfusate (1.2 mM), which would arbitrarily increase the palmitoyl CoA levels in perfused hearts from low fat fed animals. As such, palmitoyl CoA levels accounted for at least 70% of the total long chain acyl CoA content measured in all groups, explaining why no increase in long chain acyl CoA was observed in hearts following DIO in this study. High levels of palmitoyl CoA may also account for a lack of increase in intra-myocardial ceramide content, as palmitoyl CoA is the substrate for SPT I-mediated ceramide biosynthesis, and increased palmitoyl CoA levels in hearts from lean mice may also arbitrarily increase their ceramide content, masking any difference between lean and obese mice. However, our previous studies looking at ceramide content in non-perfused hearts from DIO mice also do not demonstrate an increase in ceramide content [12], [27]. Another factor that may contribute to the lack of observed increase in intra-myocardial ceramide content involves the young age of the mice in our study, as a recent study by Sung *et al.* demonstrated no change in ceramide content following DIO in young mice, but a significant increase if middle-aged mice were subjected to DIO [34]. Interestingly, we did observe an increase in intra-myocardial DAG content following DIO, which was prevented in mice treated with myriocin. This finding is of particular significance, as we have previously reported that intra-myocardial DAG accumulation is more strongly associated with the development of myocardial insulin resistance and impaired glucose utilization versus the other lipid metabolites [27]. Whether the effect of myriocin on intra-myocardial DAG content alone is responsible for improving myocardial glycolysis rates in DIO mice has not been conclusively demonstrated in this study, as myriocin treatment also resulted in a marked reduction in intra-myocardial ceramide levels. However, in *db/db* mice myriocin treatment reduced ceramide content without altering DAG levels, and had no effect on insulin signaling, supporting the premise that DAG is the more important lipid metabolite in the heart affecting cardiac insulin sensitivity and subsequent glucose utilization. Nevertheless, further characterization on the individual contributions of both ceramide and DAG towards myocardial glucose utilization is required.

The improvement in myocardial glycolysis rates in DIO mice treated with myriocin suggests an increase in myocardial glucose uptake. However, because our protocol for measuring glycolysis did not factor in the ^3^H radiolabel incorporating into glycogen, we cannot conclude for certain that glucose uptake is increased in hearts from DIO mice treated with myriocin. Despite this limitation, insulin stimulated phosphorylation of Akt, a key molecule involved in the insulin signaling cascade that regulates glucose uptake [15], [36], [37], [38], at serine 473 (indicative of increased Akt activity) is increased in DIO mice treated with myriocin. It is also important to note that while myriocin treated DIO mice exhibited an improvement in myocardial glycolytic rates, they were not normalized to normal values observed in lean mice, illustrating that lipotoxic metabolites such as ceramide and DAG are not the sole contributors to obesity-induced impairments in myocardial glucose utilization. Indeed, inflammation and oxidative stress may also impair myocardial glucose utilization [39], [40], and it is possible that while SPT I inhibition improves myocardial lipid status, it has no impact on these other mechanisms that may be activated in response to DIO.

Our previous findings have also demonstrated that DIO impairs myocardial glucose oxidation rates [12], [27], but due to the high variability in glucose oxidation rates in hearts from low fat fed animals, we did not report a decrease in this study, though there was a very strong trend to a reduction in hearts from control treated DIO mice. Further support for this observation in control treated DIO mice is evident with the 44% reduction in myocardial PDH activity, the rate-limiting enzyme for myocardial glucose oxidation. Interestingly, PDH activity was not reduced in hearts from myriocin treated DIO mice, once more suggesting that SPT I inhibition improves obesity-induced impairments on myocardial glucose utilization.

Although myriocin treatment improved myocardial glucose utilization in DIO mice, we observed a slight increase in H^+^ production in hearts from myriocin treated DIO mice, however this did not reach statistical significance. In the aerobic setting where we observed no difference in cardiac function between low fat fed and DIO mice, as such, the consequences of this H^+^ load are likely minimal. Nonetheless, as even minor changes in intracellular pH in the heart can significantly affect the recovery of cardiac function during acute metabolic stresses such as ischemia and reperfusion, the functional consequences of this slight increase in H^+^ production need to be further characterized [16], [17], [41]. Interestingly, in the presence of elevated fatty acid concentrations we have actually shown that treatment of the mouse heart with insulin decreases the recovery of cardiac function during ischemia/reperfusion, due to insulin increasing H^+^ production as a result of increasing glycolytic rates to a greater extent than glucose oxidation rates [42]. Since myriocin treatment can reverse obesity-induced insulin resistance and enhance energy metabolism in mice [43], [44], while also improving beta cell function in obese rats [45], our future and ongoing studies are addressing the potential role of SPT 1 in ischemic heart disease.

In summary, we demonstrate that DIO impairs myocardial glucose utilization, which can be improved via SPT I inhibition. The effect of SPT I inhibition was associated with a marked reduction in intra-myocardial ceramide levels and prevented the DIO-associated increase in intra-myocardial DAG content, both of which likely contribute to the improved myocardial glucose utilization. Although SPT I inhibition may represent a potential novel target for the treatment of myocardial insulin resistance, because glucose utilization was not restored to basal levels observed in lean mice, further characterization of obesity-induced myocardial insulin resistance and its associated mechanisms is required.

## References

[1] Young ME, Guthrie PH, Razeghi P, Leighton B, Abbasi S (2002). Impaired long-chain fatty acid oxidation and contractile dysfunction in the obese Zucker rat heart.. Diabetes.

[2] James PT, Leach R, Kalamara E, Shayeghi M (2001). The worldwide obesity epidemic.. Obes Res.

[3] James PT, Rigby N, Leach R (2004). The obesity epidemic, metabolic syndrome and future prevention strategies.. Eur J Cardiovasc Prev Rehabil.

[4] King H, Aubert RE, Herman WH (1998). Global burden of diabetes, 1995–2025: prevalence, numerical estimates, and projections.. Diabetes Care.

[5] International Diabetes Federation.. http://www.eatlas.idf.org/.

[6] Sharma S, Adrogue JV, Golfman L, Uray I, Lemm J (2004). Intramyocardial lipid accumulation in the failing human heart resembles the lipotoxic rat heart.. Faseb J.

[7] Wilson CR, Tran MK, Salazar KL, Young ME, Taegtmeyer H (2007). Western diet, but not high fat diet, causes derangements of fatty acid metabolism and contractile dysfunction in the heart of Wistar rats.. Biochem J.

[8] Young ME, McNulty P, Taegtmeyer H (2002). Adaptation and maladaptation of the heart in diabetes: Part II: potential mechanisms.. Circulation.

[9] Lopaschuk GD, Ussher JR, Folmes CD, Jaswal JS, Stanley WC (2010). Myocardial fatty acid metabolism in health and disease.. Physiological reviews.

[10] Atkinson LL, Kozak R, Kelly SE, Onay Besikci A, Russell JC (2003). Potential mechanisms and consequences of cardiac triacylglycerol accumulation in insulin-resistant rats.. Am J Physiol Endocrinol Metab.

[11] Finck BN, Lehman JJ, Leone TC, Welch MJ, Bennett MJ (2002). The cardiac phenotype induced by PPARalpha overexpression mimics that caused by diabetes mellitus.. J Clin Invest.

[12] Ussher JR, Koves TR, Jaswal JS, Zhang L, Ilkayeva O (2009). Insulin-stimulated cardiac glucose oxidation is increased in high-fat diet-induced obese mice lacking malonyl CoA decarboxylase.. Diabetes.

[13] Chiu HC, Kovacs A, Ford DA, Hsu FF, Garcia R (2001). A novel mouse model of lipotoxic cardiomyopathy.. J Clin Invest.

[14] Park TS, Hu Y, Noh HL, Drosatos K, Okajima K (2008). Ceramide is a cardiotoxin in lipotoxic cardiomyopathy.. J Lipid Res.

[15] Summers SA (2006). Ceramides in insulin resistance and lipotoxicity.. Prog Lipid Res.

[16] Liu B, Clanachan AS, Schulz R, Lopaschuk GD (1996). Cardiac efficiency is improved after ischemia by altering both the source and fate of protons.. Circ Res.

[17] Liu Q, Docherty JC, Rendell JC, Clanachan AS, Lopaschuk GD (2002). High levels of fatty acids delay the recovery of intracellular pH and cardiac efficiency in post-ischemic hearts by inhibiting glucose oxidation.. J Am Coll Cardiol.

[18] Dennis SC, Gevers W, Opie LH (1991). Protons in ischemia: where do they come from; where do they go to?. J Mol Cell Cardiol.

[19] Opie LH (1990). Myocardial ischemia–metabolic pathways and implications of increased glycolysis.. Cardiovasc Drugs Ther.

[20] Dyck JR, Hopkins TA, Bonnet S, Michelakis ED, Young ME (2006). Absence of malonyl coenzyme A decarboxylase in mice increases cardiac glucose oxidation and protects the heart from ischemic injury.. Circulation.

[21] Gao S, Kinzig KP, Aja S, Scott KA, Keung W (2007). Leptin activates hypothalamic acetyl-CoA carboxylase to inhibit food intake.. Proc Natl Acad Sci U S A.

[22] Bose R, Kolesnick R (2000). Measurement of ceramide levels by the diacylglycerol kinase reaction and by high-performance liquid chromatography-fluorescence spectrometry.. Methods Enzymol.

[23] Constantin-Teodosiu D, Cederblad G, Hultman E (1991). A sensitive radioisotopic assay of pyruvate dehydrogenase complex in human muscle tissue.. Anal Biochem.

[24] Ussher JR, Jaswal JS, Wagg CS, Armstrong HE, Lopaschuk DG (2009). Role of the atypical protein kinase Czeta in regulation of 5′-AMP-activated protein kinase in cardiac and skeletal muscle.. American journal of physiology Endocrinology and metabolism.

[25] Ussher JR, Lopaschuk GD (2008). The malonyl CoA axis as a potential target for treating ischaemic heart disease.. Cardiovascular research.

[26] Ussher JR, Lopaschuk GD (2009). Targeting malonyl CoA inhibition of mitochondrial fatty acid uptake as an approach to treat cardiac ischemia/reperfusion.. Basic Res Cardiol.

[27] Zhang L, Ussher JR, Oka T, Cadete VJ, Wagg C (2011). Cardiac diacylglycerol accumulation in high fat-fed mice is associated with impaired insulin-stimulated glucose oxidation.. Cardiovasc Res.

[28] Buchanan J, Mazumder PK, Hu P, Chakrabarti G, Roberts MW (2005). Reduced cardiac efficiency and altered substrate metabolism precedes the onset of hyperglycemia and contractile dysfunction in two mouse models of insulin resistance and obesity.. Endocrinology.

[29] Mazumder PK, O’Neill BT, Roberts MW, Buchanan J, Yun UJ (2004). Impaired cardiac efficiency and increased fatty acid oxidation in insulin-resistant ob/ob mouse hearts.. Diabetes.

[30] Yang J, Sambandam N, Han X, Gross RW, Courtois M (2007). CD36 deficiency rescues lipotoxic cardiomyopathy.. Circ Res.

[31] An D, Kewalramani G, Chan JK, Qi D, Ghosh S (2006). Metformin influences cardiomyocyte cell death by pathways that are dependent and independent of caspase-3.. Diabetologia.

[32] de Vries JE, Vork MM, Roemen TH, de Jong YF, Cleutjens JP (1997). Saturated but not mono-unsaturated fatty acids induce apoptotic cell death in neonatal rat ventricular myocytes.. J Lipid Res.

[33] Baranowski M, Blachnio-Zabielska A, Hirnle T, Harasiuk D, Matlak K (2010). Myocardium of type 2 diabetic and obese patients is characterized by alterations in sphingolipid metabolic enzymes but not by accumulation of ceramide.. Journal of lipid research.

[34] Sung MM, Koonen DP, Soltys CL, Jacobs RL, Febbraio M (2011). Increased CD36 expression in middle-aged mice contributes to obesity-related cardiac hypertrophy in the absence of cardiac dysfunction.. J Mol Med.

[35] Somoza B, Guzman R, Cano V, Merino B, Ramos P (2007). Induction of cardiac uncoupling protein-2 expression and adenosine 5′-monophosphate-activated protein kinase phosphorylation during early states of diet-induced obesity in mice.. Endocrinology.

[36] Farese RV, Sajan MP, Standaert ML (2005). Insulin-sensitive protein kinases (atypical protein kinase C and protein kinase B/Akt): actions and defects in obesity and type II diabetes.. Exp Biol Med (Maywood).

[37] Standaert ML, Sajan MP, Miura A, Kanoh Y, Chen HC (2004). Insulin-induced activation of atypical protein kinase C, but not protein kinase B, is maintained in diabetic (ob/ob and Goto-Kakazaki) liver. Contrasting insulin signaling patterns in liver versus muscle define phenotypes of type 2 diabetic and high fat-induced insulin-resistant states.. J Biol Chem.

[38] Choi CS, Savage DB, Abu-Elheiga L, Liu ZX, Kim S (2007). Continuous fat oxidation in acetyl-CoA carboxylase 2 knockout mice increases total energy expenditure, reduces fat mass, and improves insulin sensitivity.. Proc Natl Acad Sci U S A.

[39] Tan Y, Ichikawa T, Li J, Si Q, Yang H (2011). Diabetic downregulation of Nrf2 activity via ERK contributes to oxidative stress-induced insulin resistance in cardiac cells in vitro and in vivo.. Diabetes.

[40] Tessier JP, Thurner B, Jungling E, Luckhoff A, Fischer Y (2003). Impairment of glucose metabolism in hearts from rats treated with endotoxin.. Cardiovascular research.

[41] Ussher JR, Wang W, Gandhi M, Keung W, Samokhvalov V (2012). Stimulation of glucose oxidation protects against acute myocardial infarction and reperfusion injury.. Cardiovascular research.

[42] Folmes CD, Clanachan AS, Lopaschuk GD (2006). Fatty acids attenuate insulin regulation of 5′-AMP-activated protein kinase and insulin cardioprotection after ischemia.. Circ Res.

[43] Ussher JR, Koves TR, Cadete VJ, Zhang L, Jaswal JS (2010). Inhibition of de novo ceramide synthesis reverses diet-induced insulin resistance and enhances whole-body oxygen consumption.. Diabetes.

[44] Yang G, Badeanlou L, Bielawski J, Roberts AJ, Hannun YA (2009). Central role of ceramide biosynthesis in body weight regulation, energy metabolism, and the metabolic syndrome.. American journal of physiology Endocrinology and metabolism.

[45] Shimabukuro M, Higa M, Zhou YT, Wang MY, Newgard CB (1998). Lipoapoptosis in beta-cells of obese prediabetic fa/fa rats. Role of serine palmitoyltransferase overexpression.. J Biol Chem.

